# Molecular and phylogenetic characterization of *Cryptosporidium* species in the saffron finch *Sicalis flaveola*

**DOI:** 10.1186/s12917-022-03553-5

**Published:** 2022-12-24

**Authors:** Francisco Carlos Rodrigues de Oliveira, Samira Salim Mello Gallo, Taynara Kerolayne Santos Elizeu, Nicole Brand Ederli

**Affiliations:** 1 Laboratório de Sanidade Animal, Universidade Estadual do Norte Fluminense Darcy Ribeiro, Av. Alberto Lamego, 2000, Campos dos Goytacazes, Rio de Janeiro, 28013-602 Brazil; 2 Instituto do Noroeste Fluminense de Educação Superior, Universidade Federal Fluminense, Avenida João Jasbick, Santo Antônio de Pádua, Rio de Janeiro, 28470-000 Brazil

**Keywords:** Protozoan, *Cryptosporidium galli*, *Cryptosporidium andersoni*, Molecular biology, *Sicalis flaveola*

## Abstract

**Background:**

*Cryptosporidium* is the most common protozoan that can infect a wide variety of animals, including mammals and birds. Fecal samples of six saffron finches, *Sicalis flaveola,* from a commercial establishment were screened for the presence of *Cryptosporidium* by the modified Ziehl–Neelsen technique and nested PCR of the 18S rRNA gene followed by sequencing of the amplified fragments.

**Results:**

The species *Cryptosporidium galli* was identified in all six saffron fiches, in addition to *Cryptosporidium andersoni* in one of the birds, indicating a mixed infection. Only two birds had feathers that were ruffled and dirty with feces. Concomitant infection with *Isospora* spp. was observed in all birds.

**Conclusions:**

Saffron finches are a possible host of *C. andersoni* and this is the first report of this species in a captive bird and the third report of parasitism by *C. galli* in *Sicalis flaveola*.

## Background

Protozoa of the genus *Cryptosporidium* belong to the Phylum Miozoa, Subphylum Myzozoa, Infraphylum Apicomplexa, Superclass Sporozoa, Class Gregarinomorphea, Subclass Cryptogregaria and Order Cryptogregarida [1]. *Cryptosporidium* is one of the most important parasitic protozoa that can be transmitted through food and water contamination and is recognised as a major contributor to morbidity and is estimated to cause an annual global loss of 13 million disability adjusted life years (DALYs) [2].

*Cryptosporidium* is characterized by extensive genetic variation and pathogenicity. To date, there are 44 valid species and about 60 genotypes reported from all over the world [3–6]. There are 8 species of *Cryptosporidium* that infect birds: *Cryptosporidium meleagridis*, *Cryptosporidium baileyi*, *Cryptosporidium galli*, *Cryptosporidium ornithophilus*, *Cryptosporidium proventriculi*, *Cryptosporidium avium*, *Cryptosporidium parvum* and *Cryptosporidium andersoni* [7, 8].

*Cryptosporidium baileyi* infects the epithelium of a wide variety of organs, such as the trachea and the bursa of Fabricius, while *C. meleagridis* infects the small intestine and cecum [9, 10]. *Cryptosporidium galli* causes changes in the proventriculus as the parasite develops in the epithelial cells of this organ and does not affect either the intestines or the respiratory tract [11]. *C. ornithophilus* n. sp. infects the caecum, colon and bursa Fabricii [4]. *C. proventriculi* infects the microvilli in the proventriculus and ventriculus [12]. *C. avium* infects the microvilli in the ileum and caecum [13]. *C. parvum* develops lesions in the small intestine and cecum and can cause disruption of intestinal epithelial integrity [14, 15]. *C. andersoni* has been found in the feces of wild bird [8, 16–18] but its site of infection in birds is not yet defined.

The aim of the present study was identify and characterize molecularly *Cryptosporidium* species in fecal samples of saffron finches, *Sicalis flaveola,* from a commercial farm in the city of Campos dos Goytacazes, state of Rio de Janeiro, Brazil.

## Results

**Fig. 1:**
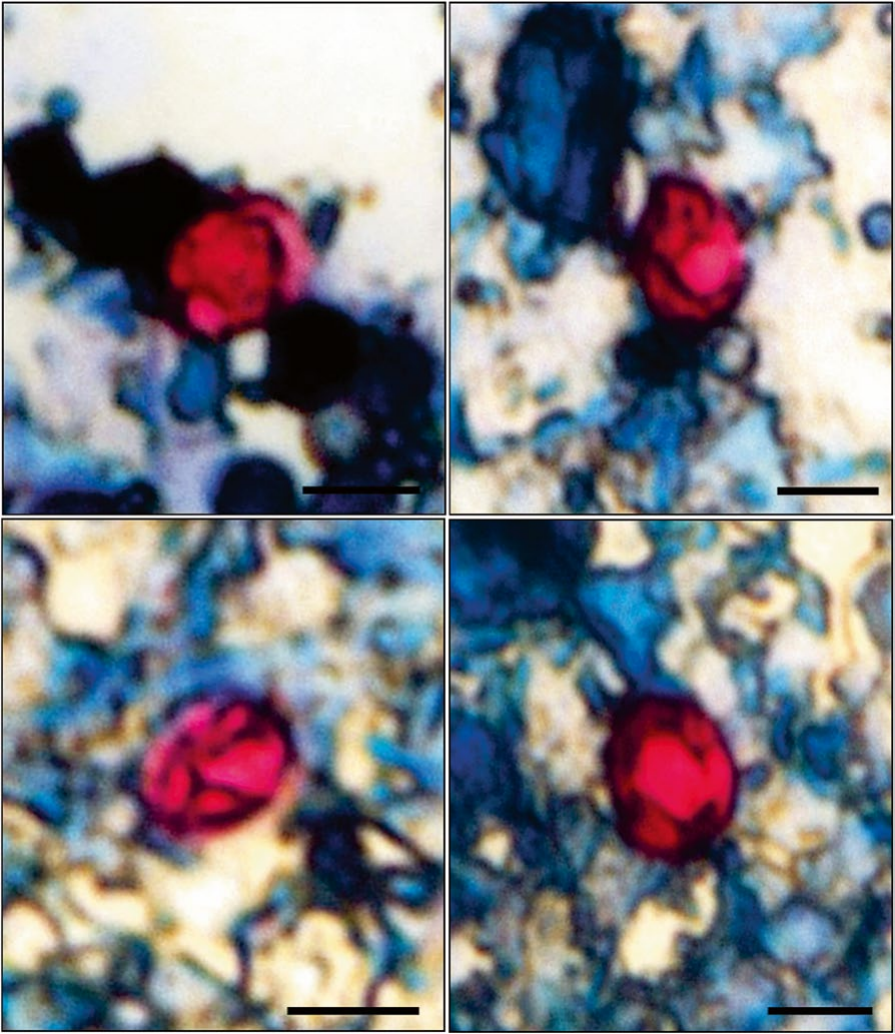
Sporulated oocysts of *Cryptosporidium galli* stained by the modified Ziehl–Neelsen technique. Bars: 5 µm

*Cryptosporidium* oocysts were detected in all fecal samples by microscopic analysis of smears stained using the Ziehl–Neelsen technique (Fig. 1).

**Fig. 2:**
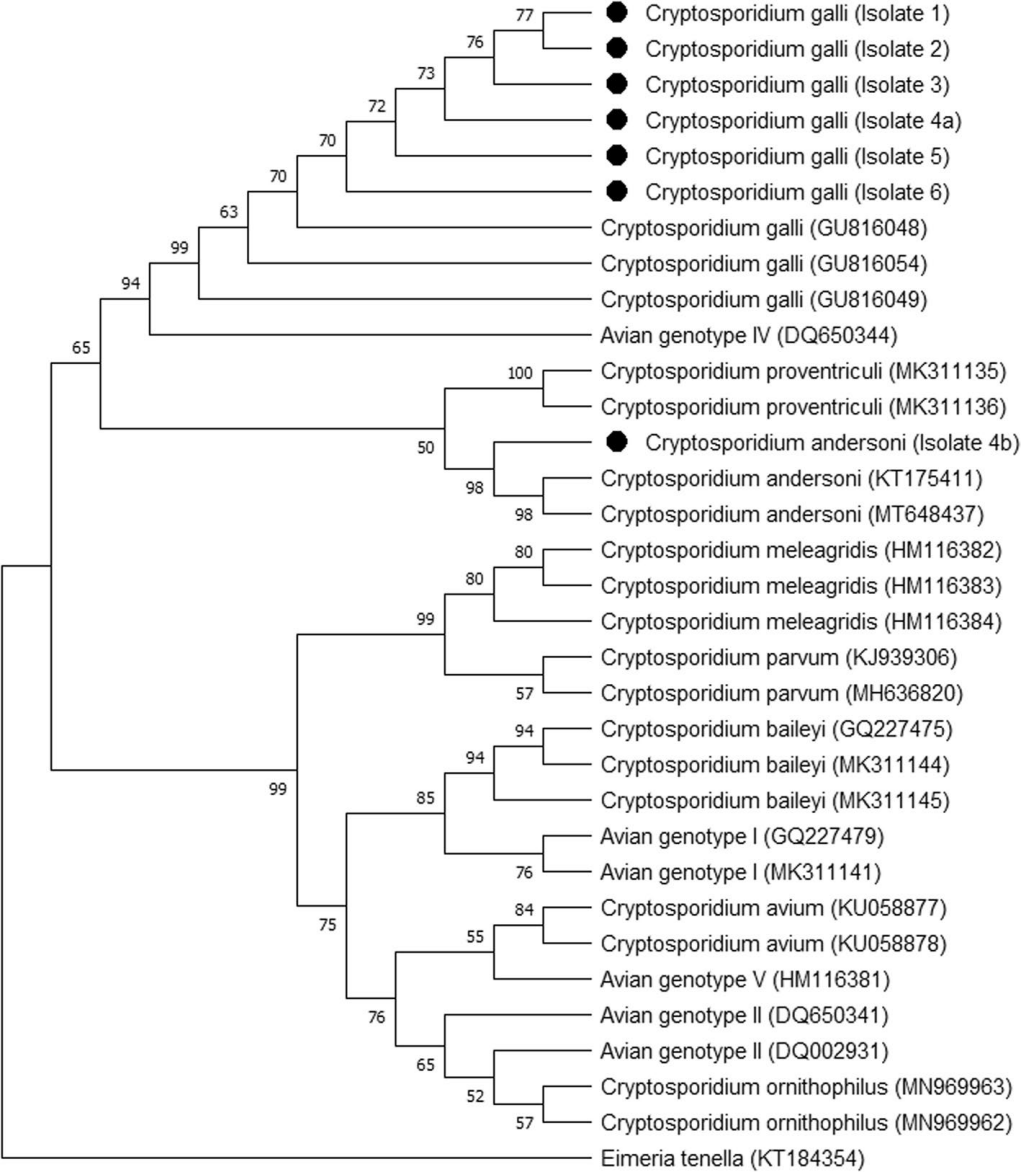
Phylogenetic analysis of *Cryptosporidium* spp. using the neighbor-Joining method and the Kimura 2-parameter model based on isolated sequences of the 18S rRNA gene of *Cryptosporidium* from this experiment and other *Cryptosporidium* species found in birds

Molecular analysis revealed amplification of *Cryptosporidium* 18S rRNA gene in all samples analyzed. By sequencing the fragment amplified by nested PCR (n-PCR), the species *C. galli* was identified in all samples, but in one of the birds, a mixed infection was detected since in one of the sequencing runs, the species *C. andersoni* was identified. The *C. galli* isolates from the present study shared 99.71–100% similarity with other *C. galli* isolates according to nBlast analysis, and the *C. andersoni* isolate from one of the birds shared 100% identity with *C. andersoni* isolated from a whooper swan (*Cygnus cygnus*). Molecular characterization of the seven *Cryptosporidium* sequences was performed with phylogenetic reconstructions of the 18S rRNA gene using a total of 343 positions in the final dataset. Phylogenetic reconstructions of the *Cryptosporidium* 18S rRNA gene sequences from *S. flaveola* can be seen in Fig. 2.

The sporulated oocysts of *C. galli* (*n* = 117) were 5.81 ± 0.78 (3.97–8.09) by 4.86 ± 0.66 (3.3–7.23) µm on average, with a length/width ratio of 1.20 ± 0.12 (0.95–1.50). The oocysts of the positive sample for two *Cryptosporidium* species were not measured. The microscopic analysis also detected oocysts of *Isospora* spp. Two birds had feathers ruffled and soiled with feces.

## Discussion

Common techniques used to diagnose *Cryptosporidium* infection are microscopic analysis and n-PCR [19]. Despite microscopy being an intensive procedure that demands time and experience, the extraction of DNA from fecal samples of *S. flaveola* was performed only by means of previous microscopy. As discussed by Nakamura et al. [20], performing PCR on samples previously identified as positive by microscopy implies a lower cost, as the reagents are expensive. Microscopy is an affordable and quick technique; however, it does not identify *Cryptosporidium* species and is less sensitive and specific. Therefore, n-PCR was performed to allow the identification of the species after amplicon sequencing.

In the present study, we identified only 2 of the 8 species of *Cryptosporidium* already found in birds. The 100% positivity of saffron finches, family Emberizidae, was high, but the number of samples collected and analyzed was low, making it difficult to compare the prevalence with that reported in most studies of *Cryptosporidium* in captive and wild birds. One factor that may interfere with the rate of *Cryptosporidium* infection is a difference in the age of the animals [21, 22], although almost all reports of *Cryptosporidium* infections in captive or wild birds do not specify the age range of the animals examined.

Silva et al. [23] carried out a study in which 480 samples of passerine feces were collected from Araçatuba, São Paulo. Of these samples, 105 were positive for *Cryptosporidium*, with n-PCR and sequencing revealing all infections to be of the species *C. galli*. Similarly, Antunes et al. [24] detected the species *C. galli* in all samples studied through molecular analysis, with four canaries (*Serinus canaria*) and eight cockatiels (*Nymphicus hollandicus*) in captivity. These works corroborate the finding of the present study in which *C. galli* was detected in all PCR-positive samples.

The mean size of *C. galli* oocysts obtained in the present study was smaller than the mean size of *C. galli* reported by Ryan et al. [25] and by Qi et al. [26]. The acid-fast staining causes shrinkage and deformation of oocysts and this could explain the smaller size of oocysts in this study as compared to other studies. Since the sizes of the oocysts of different species of *Cryptosporidium* are very similar, oocyst morphometry alone is not sufficient to distinguish the species, making molecular studies necessary for accurate identification.

Among the species/genotypes of *Cryptosporidium* in birds, only two named species, *C. baileyi* and *C. galli*, were identified in the saffron finch, *S. flaveola,* in previous studies [20, 27, 28]. Nakamura et al. [20] conducted a study of 966 stool samples from birds belonging to 18 families. These captive or wild birds came from three Brazilian states: Goiás, Paraná and São Paulo. In a specimen of *S. flaveola*, the species *C. baileyi* (GQ227475) was diagnosed through PCR and sequencing of the 18S rRNA gene. In 2012, Sevá and colleagues [27] analyzed 242 fecal samples from wild birds seized by the environmental control agency of São Paulo State. Four *S. flaveola* were positive for *Cryptosporidium,* three birds harbored the species *C. galli* (GU816048, GU816069, HM126668), and one was positive for the species *C. baileyi* (GU816042). Nakamura et al. [28] collected a total of 1027 fecal samples from birds of the orders Psittaciformes and Passeriformes. These birds were from captivity or the wild and came from Divisão Técnica de Medicina Veterinária e Manejo da Fauna Silvestre (DEPAVE-3) of São Paulo. Of the 108 positive samples, 40 were sequenced, one of which was from *S. flaveola* and was positive for *C. galli* according to n-PCR sequencing (accession number in GenBank not available). Even though our research represents the third diagnostic report of *C. galli* in *S. flaveola*, further studies are still needed on species or genotypes of *Cryptosporidium* that can infect this species of Passeriformes.

A coinfection of *C. galli* and *C. andersoni* occurred in one of the birds in the present study, although only monoinfections were previously found in *S. flaveola* [20, 27, 28]. According to Máca and Pavlásek [29], the intensive rearing of birds in breeders can be problematic, as it is associated with a large number of birds in a relatively small area, increasing the possibility of bacterial, viral and parasitic diseases and their rapid spread compared to those in wild birds.

The birds in the present study lived in separate cages but were kept in the same environment and close to each other. As reported by Nakamura et al. [28], this can result in the spread of infection through direct contact with feces or human transport of oocysts during routine management related to cleaning. In addition, the saffron finch cages were close to the cages of other bird species, which may have contributed to the interspecific spread of *Cryptosporidium* infections.

Specifically, *C. galli* infections are associated with other pathogens [30], and these associations can lead to weight loss, lameness, pelvic limb joint swelling and high mortality in captive birds [24]. Although the birds in the present study were infected with *Isospora* oocysts, they did not show any of these clinical symptoms. Due to the association of infections by *C. galli* and *Isospora* in the birds of the present study, it was not possible to determine which agent was responsible for the feathers ruffled and soiled with feces observed on two of the birds, since both infections can cause the observed characteristics. According to Cox et al. [31], in mixed infections, the burden of one or both infectious agents may be increased, that of one or both may be suppressed, or that of one may be increased and that of the other suppressed.

Passerines infected with *C. galli* can shed oocysts intermittently for 12–13 months [23, 24]. The determination of intermittent and prolonged shedding of *C. galli* oocysts in fecal samples, in addition to demonstrating that this species causes chronic infection in birds, also maintains the species between generations of birds through contact between parents and progeny. In view of this, it is necessary to adopt strict sanitary management measures to prevent the occurrence of infections in breeders, commercial establishments and nongovernmental organizations that receive apprehended wild birds.

The *C. andersoni* isolate from *S. flaveola* (Isolate 4b) clustered with the other isolates of the same species from previous studies (MT648437 and KT175411) with high (98%) bootstrap support (Fig. 2). The branch of the *C. andersoni* species clustered with the isolates of *C. galli,* which is also a gastric parasite, suggesting that these two *Cryptosporidium* species are close relatives. *Cryptosporidium andersoni* is a species found mainly in cattle and humans [32, 33] but was previously reported in the bird *Podargus strigoides* in an Australian study [17] and in an ostrich, *Struthio camelus*, from a zoo in southwestern France [18]. Similar to Ng et al. [17], we were unable to determine whether the presence of *C. andersoni* oocysts in the fecal samples of birds analyzed in the present study was due to a real infection or accidental contamination by mechanical transport, since the birds in the present study had close contact with humans. In addition, animals can also be infected indirectly after drinking water contaminated with *Cryptosporidium*. In view of the above, studies are needed to discover whether birds are natural hosts or only carriers of *C. andersoni*, since studies have already reported that a species of *Cryptosporidium* may have a wider host range than originally assumed [34].

## Conclusion

In conclusion, the high *C. galli* parasite load in all birds in this research shows that the saffron finch, *S. flaveola*, is a host of this protozoan species, although this is the third report of parasitism in this bird species. In addition, *S. flaveola* may contribute to the maintenance of intraspecific and interspecific infections in environments with large numbers of birds. This is the first report of *C. andersoni* in a captive bird and the low prevalence in our studies and the few reports of coccidia from this species in birds prevent us from inferring that this species of passerine is a good host or if it is simply a carrier of the protozoan. Further research is required to define the public health importance of *Cryptosporidium* in feces of birds.

## Material and methods

### Fecal samples and the Ziehl–Neelsen method

Fecal samples were collected from six adult saffron finches, *Sicalis flaveola*, from a commercial establishment in the city of Campos dos Goytacazes, Rio de Janeiro, Brazil. This study was approved by the Biodiversity Authorization and Information System (SISBIO) under protocol n° 78,016–1/2022 and all experimental protocols were approved by the ethics committee in the use of animals (protocol n° 523). The six birds were in separate cages, and all fecal content deposited at the bottom of the cage during a 24-h period was collected and placed in a sterile collection tube. The tubes were identified and transported in isothermal boxes with ice to the Center for Advanced Research in Parasitology of the Universidade Estadual do Norte Fluminense Darcy Ribeiro. A part of the fecal content was examined for the presence of oocysts of *Cryptosporidium* spp. by microscopy of fecal smears stained by the modified Ziehl–Neelsen technique according to Angus [35]. Measurements were made using a Zeiss AxioVision Sample Images Software and are provided in micrometers.

### DNA extraction and nested PCR

From the other part of the fecal content, genomic DNA was extracted using a DNA and tissue kit (QIAGEN) with some modifications of the manufacturer's protocol [36]. DNA samples were stored at − 20 °C, and all samples were screened for *Cryptosporidium* using n-PCR to amplify fragments of the 18S subunit of the rRNA gene [37, 38], with subsequent sequencing of amplified fragments. Primers P1: 5-TTTCTAGAGCTAATACATGCG-3, P2: 5-CCCATTTCCTTCGAAACAGGA-3 and P3: 5-GGAAGGGTTGTATTTATTAGATAAAG-3, P4: 5-AAGGAGTAAGGAACAACCTCCA-3 were used for the primary (~ 1325 bp) and secondary (~ 830 bp) reactions, respectively. The second amplification products were examined with 1% (w/v) agarose gel electrophoresis after staining with DNA Green (Solarbio, Beijing, China). In addition, *Cryptosporidium parvum* DNA was used as a positive control, and ultrapure water was used as a negative control.

### Sequencing and phylogenetic analysis

The amplified fragment (~ 830 bp) resulting from the secondary reaction of n-PCR was purified using the GFX PCR DNA Band Purification® Kit (GE Health Sciences, Champaign, IL, USA) and sequenced with the aid of DYEnamic® ET Kit Cycle Sequencing® Terminator Dye (GE Health Sciences, Champaign, IL, USA) on a MegaBACE® sequencer (GE Health Sciences, Champaign, IL, USA). Sequencing reactions were performed at least three times in both directions with the n-PCR secondary reaction primers. The consensus sequence was analyzed using CodonCode Aligner v.2.0.4 software (CodonCode Corp., Dedham, MA) and aligned with *Cryptosporidium* reference sequences published in GenBank (https://blast.ncbi.nlm.nih.gov/Blast.cgi) using MEGA X software [39] by the neighbor-joining method [40] after estimating the distance using the Kimura 2-parameter model [41]. All positions containing gaps and missing data were eliminated from the dataset (full delete option). In the construction of the phylogenetic tree, *Eimeria tenella* (KT184354) was used as an outgroup. Group confidence was assessed by bootstrap values using 1000 replicates.

The following sequences were used to construct the phylogenetic tree: MK311144 (*C. baileyi*) from *Erythrura gouldiae*, GQ227475 (*C. baileyi*) from *Sicalis flaveola*, MK311145 (*C. baileyi*) from *Carduelis psaltria*, MK311141 (Avian genotype I) from *Serinus canaria*, GQ227479 (Avian genotype I) of *Serinus canaria*, HM116381 (Avian genotype V) of *Nymphicus hollandicus*, DQ650341 (Avian genotype II) of *Eolophus roseicapilla*, DQ002931 (Avian genotype II) of *Struthio camelus*, HM116382 (*C. meleagridis*) of *Columba livia*, HM116383 (*C. meleagridis*) from *Bombycilla garrulus*, HM116384 (*C. meleagridis*) from *Streptopelia orientalis*, DQ650344 (Avian genotype IV) from *Zosterops japonica*, MK311135 (*C. proventriculi*) from *Poicephalus gulielmi*, MK311136 (*C. proventriculi*) from *Agapornisico rosellis*, GU816048 (*C. galli*) from *Sicalis flaveola*, GU816049 (*C. galli*) from *Saltator similis*, GU816054 (*C. galli*) from *Sporophila angolensis*, KT175411 (*C. andersoni*) from slaughterhouse wastewater, MT648437 (*C. andersoni*) from *Cygnus* sp., KJ939306 (*C. parvum*) from *Accipiter nisus*, MH636820 (*C. parvum*) from an unspecified bird, KU058877 (*C. avium*) from *Melopsittacus undulatus*, KU058878 (*C. avium*) from *Cyanoramphus novaezelandiae*, MN969963 (*C. ornithophilus*) from *Nymphicus hollandicus* and MN969962 (*C. ornithophilus*) isolated from *Anser anser*.

## Data Availability

The 18S rRNA gene nucleotide sequences were deposited in the GenBank database under accession numbers OM436006-OM436011 (*C. galli*) and OM491513 (*C. andersoni*).
